# Embolization of parastomal and small bowel ectopic varices utilizing a transhepatic antegrade approach: A case series

**DOI:** 10.1177/20584601221112618

**Published:** 2022-07-05

**Authors:** Ibrahim Mohammad Nadeem, Zain Badar, Victoria Giglio, Steffan Frosi Stella, George Markose, Sabarinath Nair

**Affiliations:** 1 Department of Radiology 3710 , McMaster University, Hamilton, ON, Canada; 2 Division of Vascular and Interventional Radiology 3710 , Department of Medical Imaging, University Health Network/University of Toronto, Toronto, ON, Canada; 3 Department of Radiology, 25453 Juravinski Hospital, McMaster University, Hamilton, ON, Canada; 4 Department of Surgery, 3710 McMaster University, Hamilton, ON, Canada

**Keywords:** Balloon-occluded antegrade transvenous obliteration, percutaneous transhepatic obliteration, parastomal varices, portal hypertension, small bowel varices

## Abstract

**Background:**

The ideal approach to managing parastomal and small bowel ectopic varices (EVs) is yet to be established.

**Purpose:**

To evaluate outcomes following percutaneous antegrade transhepatic venous obliteration (PATVO) in patients presenting with bleeding from parastomal or small bowel EVs.

**Material and Methods:**

A case series of 12 patients presenting with active or recurrent bleeding from parastomal or small bowel EVs who underwent 17 PATVO interventions at our tertiary care institution was performed. Data extraction from electronic medical records included baseline characteristics and procedural details. Endpoints included technical success, early clinical success, and re-bleeding.

**Results:**

Technical success was 100% (*n* = 17), and early clinical success was 82.3% (*n* = 14). No patient experienced any intra- or post-operative complications. Rebleed rates after initial PATVO in patients who achieved early clinical success was as follows: 3-month, 0% (*n* = 0); 6-month, 20% (*n* = 2); 12-month, 20% (*n* = 2). Rebleed rates after all PATVO procedures (including patients undergoing repeat procedures) that achieved early clinical success were as follows: 3-month, 0% (*n* = 0); 6-month, 14% (*n* = 2; 12-month, 14% (*n* = 2). All patients with re-bleeding required reintervention with either PATVO, transjugular intrahepatic portosystemic shunt (TIPS) or both.

**Conclusion:**

PATVO can be safely performed to treat bleeding from parastomal and small bowel EVs. In patients who present with recurrent bleeding despite PATVO, TIPS with/without embolization of bleeding varices remains a valid option as described by the literature.

## Introduction

Parastomal ectopic varices (EVs) are abnormally dilated mesenteric varices that develop at the mucocutaneous border of a stoma associated with ileostomies or colostomies.^1–5^ Common clinical scenarios are in patients with ileostomies after proctocolectomy for inflammatory bowel disease, in patients with portal hypertension, adhesions and scarring due to stoma creation, or surgical alterations in anatomy.^4^ Bleeding is the main presentation of parastomal and small bowel EVs, with reported mortality rates as high as 40%.^4,6,7^ Conservative management methods, including single-digit compression and epinephrine-soaked gauze, are often used for focal parastomal variceal bleeding and may be effective at immediate control. Mortality rates from conservative management for parastomal and small bowel EVs is reported to be 3–4%.^1,2^ However, in patients with oozing venous bleeding secondary to underlying portal hypertension, or patients with uncontrolled or recurrent bleeding where conservative techniques have failed, endovascular techniques, such as a transjugular intrahepatic portosystemic shunt (TIPS) with or without embolization and transvenous obliteration of the parastomal and small bowel EVs via portal venous access, can be considered as a management option.^4,7^

The ideal approach to managing parastomal and small bowel EVs is yet to be established. Transvenous obliteration with or without TIPS may be performed to manage variceal bleeding.^4^ There are varying approaches for transvenous obliteration of EVs including balloon-occluded antegrade transvenous obliteration (BATO).^4,6,8,9^ BATO refers to three technical approaches: (1) percutaneous antegrade transhepatic venous obliteration (PATVO) with or without balloon occlusion (the first described approach used for obliteration); (2) trans-TIPS obliteration; and (3) trans-iliocolic vein obliteration.^8^ TIPS has been effective in resolution of bleeding from parastomal and small bowel EVs in 60–90% of patients with portal hypertension when used alone, and in 75–95% of patients when combined with percutaneous transvenous embolization, with re-bleed rates between 17 and 31%.^4,6,7^ However, due to the invasive nature of TIPS, it has been found to be associated with high rates of procedure-related complications and can lead to hepatic encephalopathy.^6,10^ It is therefore warranted to investigate safer, less invasive approaches in the management of variceal bleeding. Here, we present our experience with, and describe the clinical outcomes of patients with bleeding from parastomal or small bowel EVs that solely underwent PATVO.

## Methods

**Figure 1.**
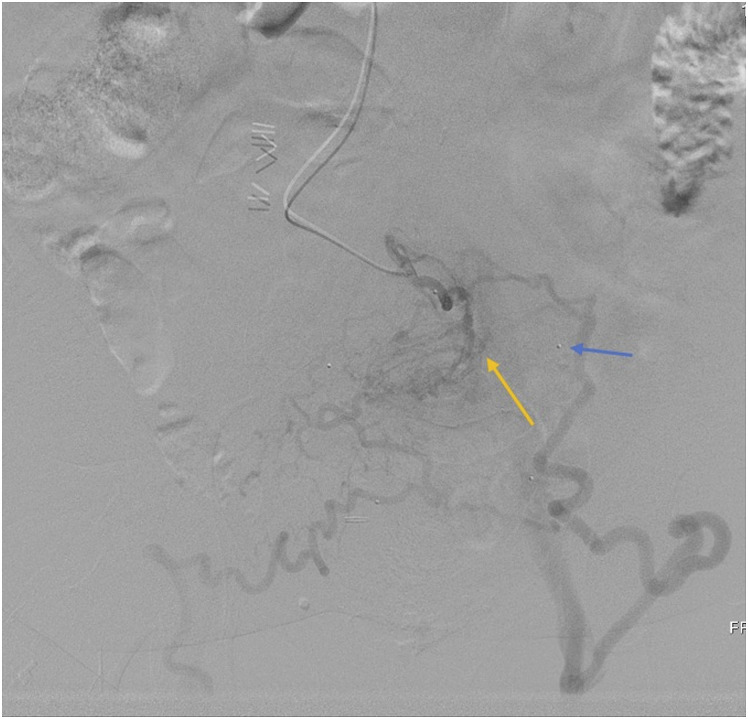
Case 1: Parastomal varix in 47-year-old female. Right portal venous access was performed with subsequent selection of an SMV branch demonstrating stomal varices (yellow arrow) in the region of stoma, identified with stomal markers (blue arrow).

**Figure 2.**
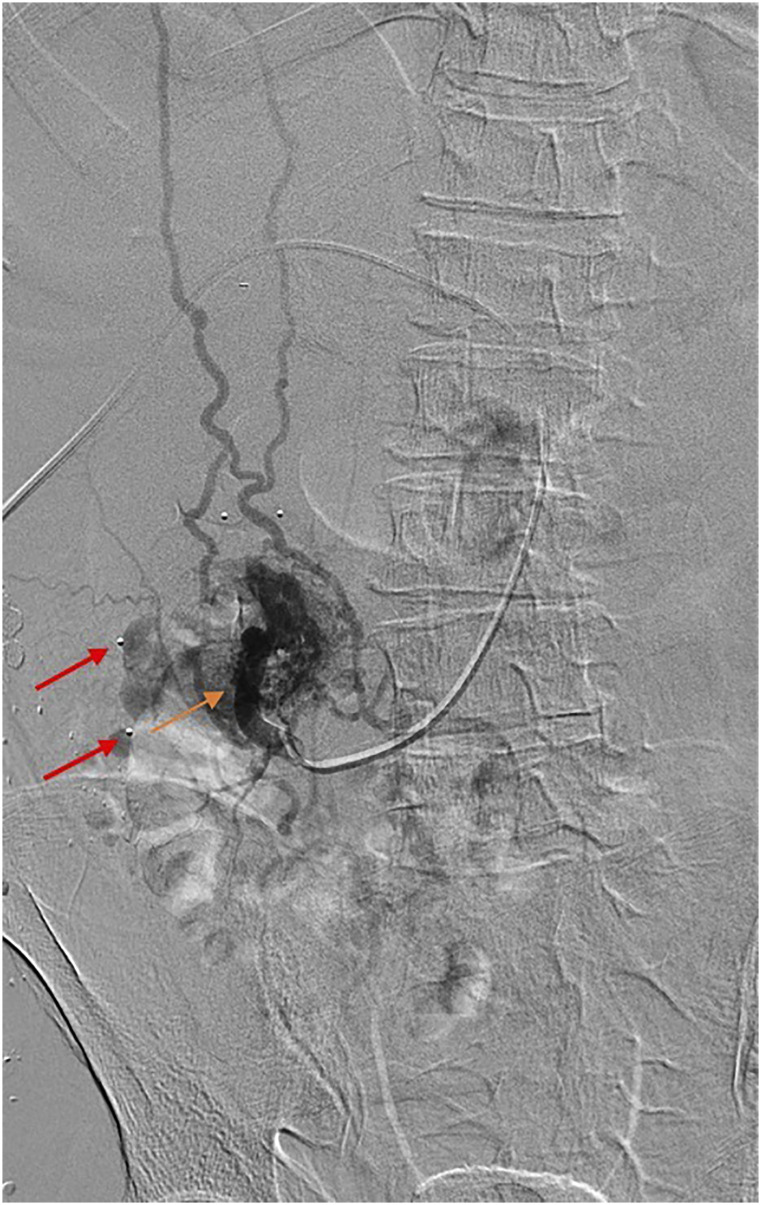
Case 2: Parastomal varix in 61-year-old male. Right portal venous access with sub-selective angiogram of a branch from the SMV supplying parastomal varices (orange arrow), with the stoma outlined via radiopaque markers (red arrow).

All consecutive patients ≥18 years of age that underwent a PATVO intervention alone for parastomal or small bowel EVs between September 2016 and September 2021 at our institution are included in this case series. Bleeding was confirmed clinically as well as via imaging (Figures 1 and 2). The use of a PATVO procedure to treat the bleeding EV was based on the interventional radiologist’s clinical decision in accordance with standard treatment at our institution. All PATVO procedures were performed by three interventional radiologists with >5 years of experience. Conscious sedation was the standard sedation used for all PATVO procedures unless general anesthesia was otherwise indicated to ensure patient comfort. Specific embolization agents utilized for each patient were chosen based on the expertise of the interventional radiologist performing the procedure. Details of the PATVO procedures performed are described in Supplementary Appendix 1. The review was approved by the institutional review board of the authors’ hospital, and the requirement for individual informed consent was waived.

Data extraction from electronic medical records included baseline characteristics, and procedural details. Endpoints were technical success, early clinical success, complications, re-bleeding, need for reintervention, and mortality. In line with previous reviews, technical success was defined as absence of contrast opacification upstream of the most downstream embolization site or resolution of blood flow in the varices on angiography immediately after embolization.^11^ Early clinical success was defined as resolution of parastomal or small bowel EVs bleeding as indicated by clinical symptoms from 0 to 30 days after procedure. Re-bleeding was defined as clinically significant re-bleeding from the primarily embolized EV only after early clinical success was achieved. Day of procedure was recorded as day 0. Descriptive statistics, percentages and means, were used to summarize categorical and continuous variables, respectively.

## Results

A total of 12 patients who underwent 17 PATVO interventions for parastomal or small bowel EVs between September 2016 and September 2021 were identified. Patient demographics and EV anatomical data are described in Supplementary Appendix 2.

Seventeen PATVO interventions were performed. PATVO was performed on an emergent basis for 11 procedures (65%) and as an elective procedure for the remaining six (35%) (Table 1). Conscious sedation, using midazolam and fentanyl, was used in 59% of cases (*n* = 10). General anesthesia was used for 29% of cases (*n* = 5), and deep sedation, provided by anesthesiologist, for 12% (*n* = 2).

**Table 1.** Procedural data.

Case	Acuity	Procedural sedation	Approach	Coils	Embozene^®^ particles	Thrombin	NBAC
Mode	Urgent	Conscious sedation	Trans-hepatic portal venous access	No	No	No	Yes
1	Urgent	Deep sedation	Trans-hepatic portal venous access	No	Yes	Yes	No
2	Urgent	Conscious sedation	Trans-hepatic portal venous access	Yes	Yes	Yes	No
3	Routine	Conscious sedation	Trans-hepatic portal venous access	Yes	No	Yes	No
4	Routine	Conscious sedation	Trans-hepatic portal venous access	Yes	Yes	Yes	No
5	Urgent	Conscious sedation	Trans-hepatic portal venous access	Yes	No	Yes	No
5^a^	Urgent	Conscious sedation	Trans-hepatic portal venous access	No	No	No	Yes
6	Urgent	Conscious sedation	Trans-hepatic portal venous access	Yes	Yes	No	No
6^a^	Urgent	Conscious sedation	Trans-hepatic portal venous access	No	No	No	Yes
**7**	Routine	General anesthesia	Trans-hepatic portal venous access	Yes	No	No	Yes
7^a^	Routine	General anesthesia	Trans-hepatic portal venous access	No	No	No	Yes
8	Urgent	Conscious sedation	Trans-hepatic portal venous access	No	No	No	Yes
8^a^	Urgent	Conscious sedation	Trans-hepatic portal venous access	No	No	No	Yes
9	Urgent	General anesthesia	Trans-hepatic portal venous access	Yes	No	No	Yes
10	Urgent	Conscious sedation	Trans-hepatic portal venous access	No	No	No	Yes
10^a^	Routine	Deep sedation	Trans-hepatic portal venous access	No	No	No	Yes
11	Urgent	General anesthesia	Trans-hepatic portal venous access	No	No	No	Yes
12	Routine	General anesthesia	Trans-hepatic portal venous access	No	No	No	Yes

Abbreviation: NBAC: N-butyl Cyanoacrylate.

^a^Patients 5, 6, 7, 8, and 10 underwent repeat PATVO interventions within the review time frame.

**Figure 3.**
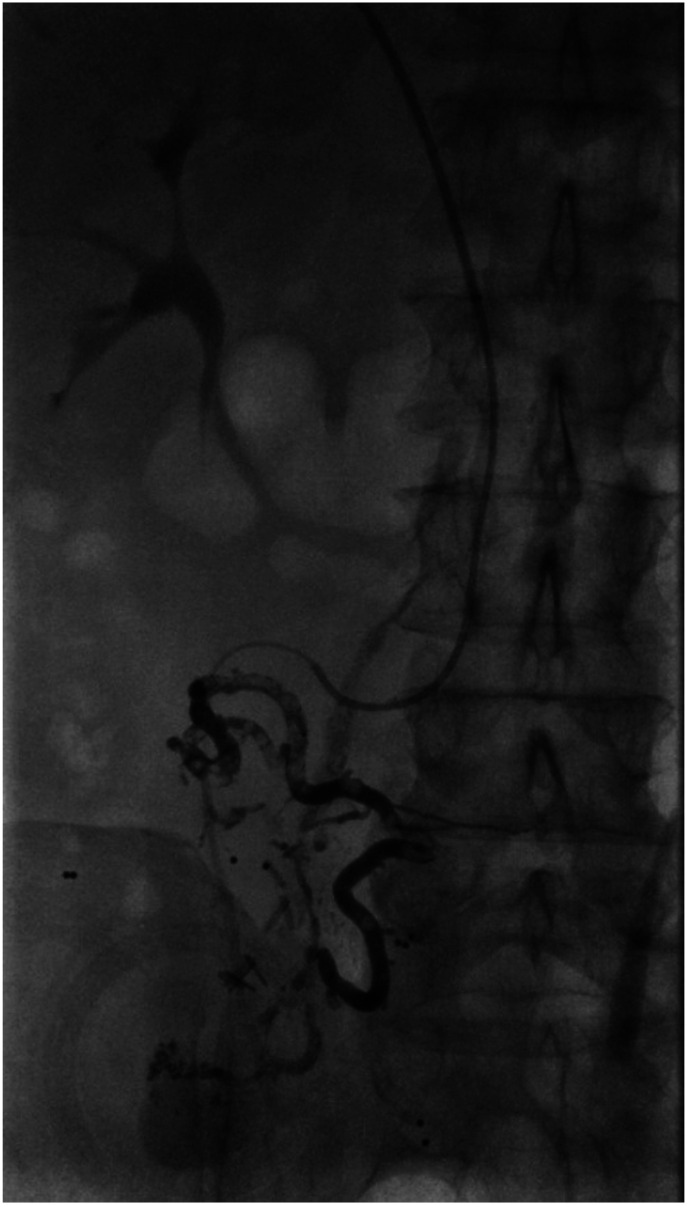
Case 1: Parastomal varix in 47-year-old female. Utilizing a 2.8 french progreat micro catheter (via a C2 glide catheter) embolization of the targeted stomal varices was performed with Glubran (cyanoacrylate glue) combined with lipiodol (1:4 ratio glubran to lipiodol). Total of 0.5 cc of Glubran was administered.

**Figure 4.**
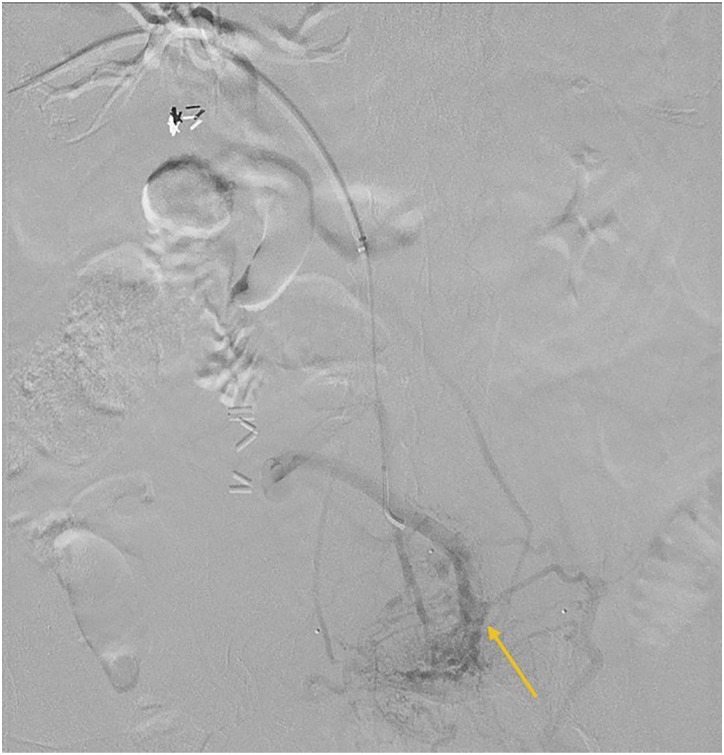
Case 1: Parastomal varix in 47-year-old female. Embolization of the SMV branch supplying stomal varices (yellow arrow) via an angled catheter was performed. Embolization agents used: embozene particles (700 μm) followed by 1000 units of thrombin.

Embolization was performed in all cases via the percutaneous antegrade transhepatic portal venous approach. Various combinations of Embozene® particles, thrombin augmentation, coils, and NBCA were employed at the discretion of the interventional radiologist as described in Supplementary Appendix 1 (Table 1). N-butyl cyanoacrylate (NBCA) alone was used in nine cases (53%) (Figure 3), thrombin augmentation and coil embolization were used in two cases (12%), Embozene® particles, thrombin augmentation and coil embolization in two cases (12%), coil embolization and glubran in one case (6%), Embozene® particles and thrombin augmentation in one case (6%) (Figure 4), Embozene® particles and NBAC in one case (6%), and Embozene® particles and coil embolization in one case (6%).

**Figure 5.**
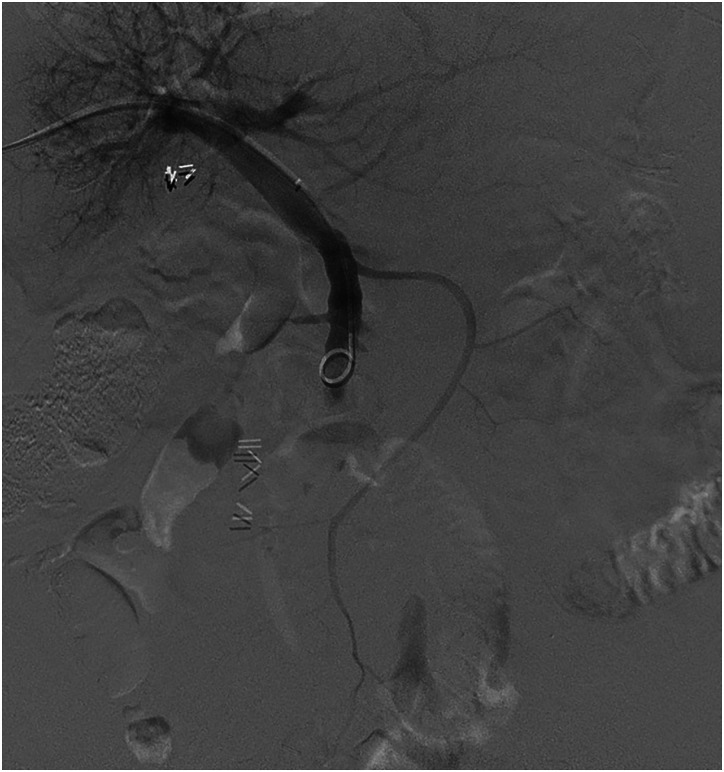
Case 1: Parastomal varix in 47-year-old female. Post embolization venogram performed via a 5F pigtail catheter within the SMV demonstrates interval resolution of parastomal varices.

**Figure 6.**
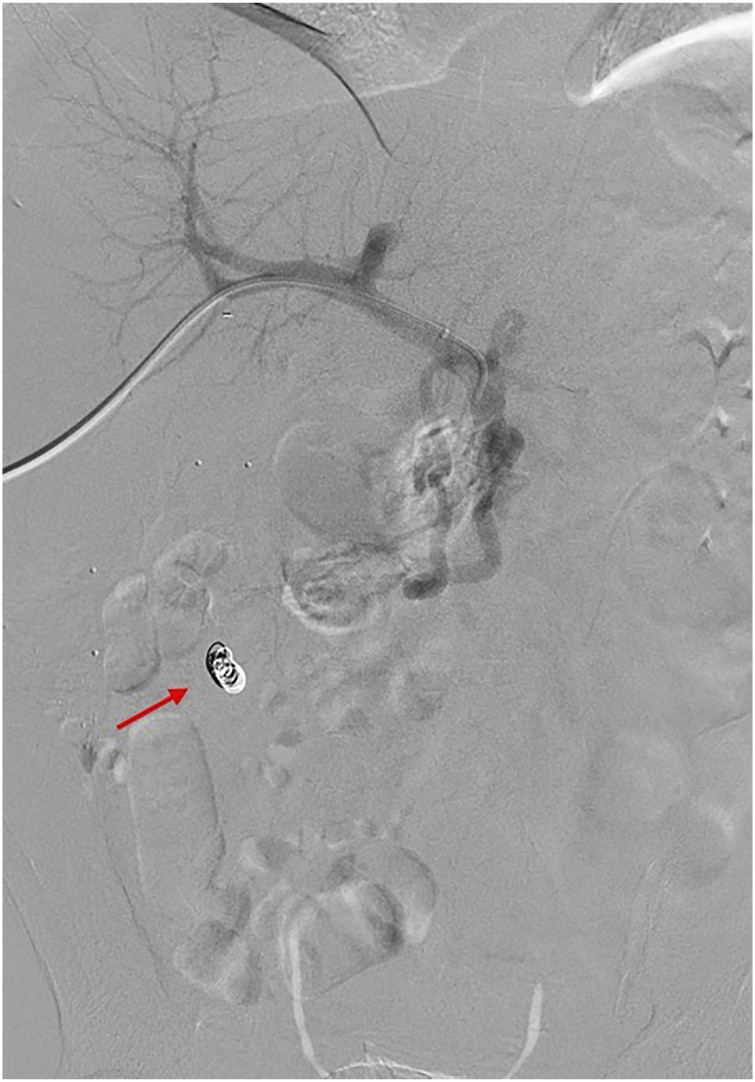
Case 2: Parastomal varix in 61-year-old male. Post embolization venogram via the SMV demonstrating interval resolution of parastomal varices. Embolization agents used: coils (red arrow), embozene particles (700 μm), and 1000 units of thrombin.

Technical success was achieved in every procedure (100%), and early clinical success was achieved in 14 procedures (82%) (Table 2) (Figures 5 and 6). One patient did not achieve early clinical success as they presented with variceal bleeding 12 days after the initial PATVO procedure. A CT scan was performed and identified an additional branch of superior mesenteric vein (SMV) supplying the varix that was not optimally visualized in the initial pre-planning CT or the intra-procedural angiogram and thus was not embolized in the initial PATVO procedure. Thus, an additional PATVO procedure was performed 15 days after the initial intervention in order to embolize the above-mentioned adjacent feeder vessel. The reintervention was technically successful with no intra- or post-operative complications and achieved early clinical success. No patient experienced any intra- or post-operative complications.

**Table 2.** Follow-up and endpoints.

Case	Technical success	Intra-operative complication	Post-operative complication	Early clinical success	Follow-up interval, days	Recurrent variceal bleeding	Time interval to re-bleeding, days	Need for reintervention	Type of reintervention	Time interval to reintervention, days	All-cause mortality
1	Yes	No	No	Yes	721	Yes	178	Yes	TIPS	283	3 years after initial PATVO - Due to unspecified comorbidities
2	Yes	No	No	Yes	LTFU	LTFU	LTFU	LTFU	LTFU	LTFU	LTFU
3	Yes	No	No	Yes	LTFU	LTFU	LTFU	LTFU	LTFU	LTFU	LTFU
4	Yes	No	No	Yes	702	Yes	371	Yes	TIPS	602	None
5	Yes	No	No	Yes	1157, then transferred to another hospital for liver transplant	Yes	NR	Yes	PATVO	1110	3 years after initial PATVO – Due to hepatic encephalopathy complications
5^a^	Yes	No	No	No	1157, then transferred to another hospital for liver transplant	Yes	1	NR	NR	NR	3 years after initial PATVO – Due to hepatic encephalopathy complications
6	Yes	No	No	No	12	No^b^	N/A	Yes^b^	PATVO	15	None
6^a^	Yes	No	No	Yes	153 (168 total)	No	N/A	N/A	N/A	N/A	None
7	Yes	No	No	Yes	555	Yes	NR	Yes	PATVO	378	None
7^a^	Yes	No	No	Yes	555	Yes	NR	Yes	TIPS	175 days post second PATVO (553 post initial procedure)	None
8	Yes	No	No	Yes	99	Yes	99	Yes	PATVO	102	None
8^a^	Yes	No	No	Yes	96 (198 total)	No	N/A	N/A	N/A	N/A	None
9	Yes	No	No	Yes	316	No	N/A	N/A	N/A	N/A	None
10	Yes	No	No	No	37	Yes	8	Yes	PATVO	37	5 months after initial PATVO – Due to progression of cancer
10^a^	Yes	No	No	Yes	0	No	N/A	N/A	N/A	N/A	5 months after initial PATVO – Due to progression of cancer
11	Yes	No	No	Yes	44	No	N/A	N/A	N/A	N/A	None
12	Yes	No	No	Yes	26	No	N/A	N/A	N/A	N/A	None

Abbreviations: N/A, not applicable; NR, not reported; LTFU, Lost to follow-up; PATVO, percutaneous antegrade transhepatic venous obliteration; TIPS, transjugular intrahepatic portosystemic shunt.

^a^Patients 5, 6, 7, 8, and 10 underwent repeat PATVO interventions within the review time frame.

^b^This patient required an additional PATVO due to an additional branch of SMV supplying the varix that was not embolized in the initial PATVO. The repeat procedure was not due to a re-bleed.

**Figure 7.**
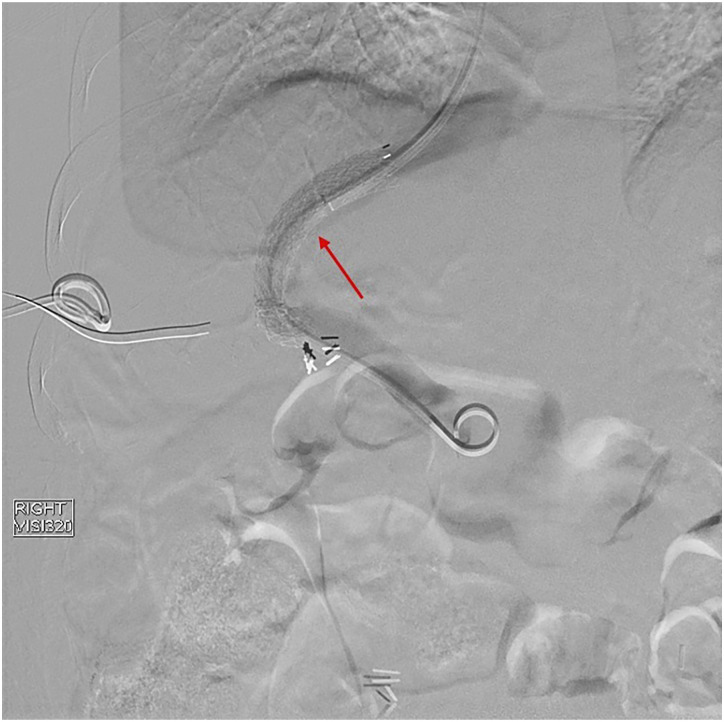
Case 1: Parastomal varix in 47-year-old female. Patient presented with parastomal variceal re-bleeding after 178 days. Subsequently, a transjugular intrahepatic portosystemic shunt stent (red arrow) was placed resulting in interval resolution of parastomal variceal bleeding.

Of the 12 initial PATVO procedures, 10 procedures achieved early clinical success. Rebleed rates after initial PATVO in patients who achieved early clinical success (*n* = 10) were as follows: 3-month rebleed rate, 0% (*n* = 0); 6-month rebleed rate, 20% (*n* = 2); and 12-month rebleed rate, 20% (*n* = 2). Rebleed rates after all PATVO procedures (including patients undergoing repeat procedures) that achieved early clinical success (*n* = 14) were as follows: 3-month rebleed rate, 0% (*n* = 0); 6-month rebleed rate, 14% (*n* = 2); and 12-month rebleed rate, 14% (*n* = 2). The time interval to re-bleeding was not reported for two patients (three procedures); however, the reintervention for these patients was >12 months post-intervention. Of the patients that re-bled following initial PATVO procedure, either from not obtaining early clinical success or from re-bleeding >30 days post-PATVO (*n* = 6), two patients underwent subsequent TIPS intervention (Figure 7), and four patients underwent subsequent PATVO. Among the patients that underwent a repeat PATVO, two patients re-bled. Of these patients, one underwent a subsequent TIPS procedure, and one patient was transferred to another hospital and expired.

Among the 12 patients included in this case series, three patients expired (25%). One patient expired 3 years after the initial PATVO intervention due to unspecified comorbidities, one patient expired 3 years after the initial PATVO intervention due to hepatic encephalopathy complications and one patient expired 5 months after the initial PATVO intervention due to progression of cancer. Two patients were lost to follow-up (16.7%). No evidence of procedure related mortality was reported with respect to any case **(**Table 2).

## Discussion

The optimal management strategy for parastomal and small bowel EVs is yet to be established. Conservative local therapies, including single-digit compression, epinephrine-soaked gauze, gel foam, and suture litigation are effective at acute control of EVs and may be the first approach at management.^12^ However, local methods are ineffective for long-term control of EVs and recurrent bleeding is expected.^7^ Conte el al. reported that 98% of patients with bleeding from EVs managed with intravenous fluids, blood transfusions, and conservative local therapies experienced recurrent bleeding within 2–10 months of management.^13^ Therefore, although conservative local therapies are considered simple and effective for acute control of bleeding from EVs, they are an ineffective for long-term management.

In patients experiencing parastomal bleeding, endovascular techniques including TIPS, transvenous obliteration such as PATVO with or without decompressive TIPS, and parastomal embolization can be considered.^4^ In the setting of a variceal bleed with generalized oozing due to congestion secondary to portal hypertension, TIPS is considered an alternative therapeutic intervention.^4^ TIPS may effectively reduce portal hypertension and has been shown to resolve hepatic congestion and bleeding, but it does not ensue without complications. Furthermore, the results in the literature highlight the possibility that transvenous obliteration procedures may be as effective as TIPS in preventing re-bleeding from varices. A systematic review of 210 patients found that 20% of patients with parastomal varices managed with TIPS alone re-bled.^14^ Similarly, Saad et al. calculated rebleed rates to be 31%, 31%, and 40% at 1, 3, and 6 months post-TIPS, respectively. Likewise, a multi-center cohort study revealed that most re-bleeds occurred within 1 month of TIPS creation.^15^ Comparatively, Saad et al. calculated the rebleed rates to be 17–31% after transvenous obliteration alone.^6^ Moreover, bleeding peristomal varices managed with a percutaneous parastomal approach does not proceed without complications. A case series reported by Pabon-Ramos et al. revealed technical challenges in a percutaneous parastomal approach of direct embolization including problems cannulating the hairpin bend between the peri-stomal varix and the portal inflow vein which ultimately lead to technical failure, as well as persistent vasospasm requiring embolization through a second access site; yet, both challenges are avoided via an anteriograde approach.^16^ In addition, this case series revealed rebleed rates of 38% at a median time of 45 days following a percutaneous parastomal.^16^ Comparatively, at 3 months, our initial PATVO rebleed rate was 0%, and 20% at 6 months post-PATVO and all procedures in the present study achieved technical success. This demonstrates that the PATVO procedure may be effective in preventing bleeding from ectopic varices compared to TIPS percutaneous parastomal approaches. Nevertheless, the rebleed rate following the management of EVs by both TIPS and transvenous obliteration procedures is relatively high and an opportunity remains for improvement in all techniques.^10,17^

Previous case reports and reviews of the literature have also indicated high rates of complications, including hepatic encephalopathy (HE), and mortality associated with TIPS procedures.^10^ Oey et al. reported that HE worsened in 30% of patients undergoing TIPS procedures.^15^ Even more concerning, a meta-analysis calculated the mortality rate at 3–6 months post-TIPS to be as high at 60%.^6^ An intra-institutional study comparing TIPS and transcatheter sclerotherapy alone for gastric varices revealed that the 1, 3, and 5 year survival after transcatheter sclerotherapy procedures was significantly better compared to TIPS procedures (96%, 83%, 76% versus 81% 64%, 40%, respectively (*p* = .01)).^18^ While this study cannot be directly compared to our EV patients undergoing PATVO, the aforementioned study does reveal strong evidence that transvenous obliteration procedures have improved survival rates compared to TIPS procedures in the management of varices. No major complications or procedure related mortality was observed in any of the included cases in our case series. Therefore, the PATVO procedure described in our case series can be considered a safe approach with respect to procedure-related mortality for the management of parastomal or small bowel EVs.

Due to the invasive nature of TIPS procedures and the relatively higher procedure related mortality and complications, TIPS is not recommended as primary prophylaxis and is only recommended as a primary technique in high-risk patients after the first variceal bleed.^4,10,19^ Furthermore, not all patients are ideal candidates for TIPS procedures due to previous abdominal surgery, pulmonary hypertension, poor hepatic reserve, and/or liver disease/portal hypertension in the setting of inflammatory bowel disease.^3,10^ Thus, as demonstrated in this case series and supported by the literature, PATVO can be considered a possible first line management option prior to TIPS. In cases of re-bleeds, either a repeat PATVO or a TIPS can be considered. However, further prospective trials are required to determine the superiority of PATVO to TIPS as the primary management option of parastomal and small bowel EVs.

Following endovascular management of bleeding EVs, re-bleeding remains a concern. A recent systematic review suggested an algorithm of the management of patients with continued bleeding from EVs, which utilized MELD scores.^12^ If the MELD score is less than or equal to 12, TIPS is recommended, and in patients with decompensated liver failure and bleeding from EVs whose MELD score is greater than 15, the patient should be evaluated for liver transplantation. Yet, transvenous obliteration techniques such as the one described within this case series were not evaluated in Spier et al.’s review or considered in the algorithm. Based on our findings, transvenous obliteration interventions are safe and effective management options and can be considered as a possible first line technique. Moreover, based on our experience and a review of the literature, we endorse a re-attempt PATVO prior to considering TIPS if the patient requires further intervention following a rebleed. We rationalize this approach due to the lower mortality rates and complications associated with transvenous obliteration procedures compared to TIPS procedures previously expressed. Within our case series, of the five patients that underwent a second attempt PATVO only two patients presented with re-bleeding at the last recorded follow-up. However, due to our limited sample size and the limited data in the literature describing outcomes following second attempt transvenous obliteration procedures, conclusive recommendations cannot be made.

If bleeding persists and cannot be controlled by the safer PATVO approach, TIPS procedure should be considered.^20^ Moreover, recent literature has advocated for TIPS plus embolization as an ideal management option in the management of recurrent bleeding from EVs, which can be considered following initial PATVO.^19,20^ A study conducted by Vangeli et al. revealed that TIPS plus embolization performed better compared to TIPS alone when evaluating rebleed rates following the management of EVs (28% versus 42%, respectively).^21^ No patient within our case series underwent a TIPS plus embolization procedure; however, we do recognize the benefits of adding embolization to TIPS due to the high rebleed rates in each of these procedures alone. While the sample sizes in the aforementioned study is small and definitive recommendations cannot be drawn, the evidence suggests the TIPS plus embolization may be beneficial in patients with recurrent EV bleeding. Nevertheless, complications including non-target coil embolization eroding into the lumen of the stoma have been reported for TIPS plus embolization procedures. Therefore, further investigation into major complication and mortality rates for TIPS plus embolization procedures is required.^4,7^

This case series presents with a few limitations. A major limitation was the small sample size and use of a variety of embolic agents. The use of different embolization agents has evolved over time, with our center’s preferred agent being Glubran, however, the effectiveness of various embolization agents could not be evaluated within this case series due to the small subgroups. Additionally, follow-up times were variable, and two patients were lost to follow-up. This case series suggests that PATVO for parastomal or small bowel EVs is a safe technique with immediate control of bleeding. Nonetheless, larger studies and randomized controlled trials comparing management approaches are warranted for definitive conclusions. Further research is also required to determine if this approach can be generalized to other varices such as gastro-esophageal varices.

In conclusion, this case series demonstrated that percutaneous transhepatic antegrade approach for embolization of bleeding from parastomal and small bowel EVs is a safe technique. Given the high technical success rate, satisfactory early clinical success, and low complication and mortality rates, PATVO can be considered as a first line management option prior to attempting TIPS. In patients who present with recurrent bleeding despite PATVO, TIPS with/without embolization of bleeding varices remains a valid option as described by the literature, however, powered randomized controlled trials are required to determine the ideal management of parastomal and small bowel EVs.

## Supplemental Material

Supplemental Material - Embolization of parastomal and small bowel ectopic varices utilizing a transhepatic antegrade approach: A case series Click here for additional data file. Supplementary Material for Embolization of parastomal and small bowel ectopic varices utilizing a transhepatic antegrade approach: A case series by Ibrahim Mohammad Nadeem, Zain Badar, Victoria Giglio, Steffan Frosi Stella, George Markose, and Sabarinath Nair in Acta Radiologica Open.
