# Supplementary material for: Embolization of parastomal and small bowel ectopic varices utilizing a transhepatic antegrade approach: A case series
Source: Acta Radiol Open. 2022 Jul 5;11(7):20584601221112618. doi: 10.1177/20584601221112618 (PMC9272059; doi:10.1177/20584601221112618)
Supplement: Supplemental Material - Embolization of parastomal and small bowel ectopic varices utilizing a transhepatic antegrade approach: A case series [file sj-pdf-1-arr-10.1177_20584601221112618.pdf]

Supplementary Appendix 1

Technique: Percutaneous Antegrade Transhepatic Venous Obliteration (PATVO)

Prior to initiation of the procedure, in order to localize parastomal or small bowel EVs during the angiography, round radiopaque markers were placed externally around the stoma site. Preliminary CT scan was utilized to aid in identifying the bleeding site, and thus the markers were placed in the region of interest (**Figure 1, Figure 2).** Using ultrasound guidance, a 21-gauge Chiba (Cook Medical, Bloomington, IN, USA) needle was used to access a peripheral segment 5 or 6 portal vein branch. A 0.018-inch cope mandril wire (Cook Medical, Bloomington, IN, USA) was introduced into the portal venous system, and the needle was removed. Over the wire, a MAK-NV coaxial introducer sheath (Merit Medical Inc., Jordan, UT, USA) was placed. The inner sheath and wire were then removed, and via the outer sheath, a portal venogram was performed. An extra stiff Amplatz (Cook Medical, Bloomington, IN, USA) guidewire was introduced via the outer sheath of the MAK-NV (Merit Medical Inc., Jordan, UT, USA) with subsequent removal of the outer sheath. Over the extra stiff Amplatz guidewire, a 5 French Kumpe catheter (Cook Medical, Bloomington, IN, USA) was introduced. The Amplatz guidewire was removed and venogram was performed via the catheter, to delineate the superior mesenteric vein (SMV) and branch points of associated varices. A combination of the Kumpe catheter and an angled 0.035 hydrophilic coated guidewire (Terumo Medical Corporation, Somerset, NJ, USA) were used to negotiate the SMV.

Following this, the hydrophilic guidewire was exchanged for an extra stiff Amplatz wire. The Kumpe catheter was then removed. Over the Amplatz wire, a 5 French long sheath (25 cm) (Terumo Medical Corporation, Somerset, NJ, USA) was positioned with the tip in the proximal SMV. Subsequently, a 5 French C2 glide catheter (Cook Medical, Bloomington, IN, USA) and 0.035 angled hydrophilic guidewire were used to access a branch of the SMV noted to provide venous drainage to the stoma on the preliminary CT scan. The radiopaque markers surrounding the stoma site were utilized as a reference point. A 2.8F progreat microcatheter (Terumo, Somerset, NJ, USA) and double angle GT 0.018-inch glidewire combination (Terumo, Somerset, NJ, USA) were used to sub-select the bleeding varix. Embolization was performed via the microcatheter (**Figure 2)**.

Different embolization agents were used for embolization based on the expertise of the interventional radiologist performing the procedure. The following embolic agents were used:

1. Coils;
2. Embozene particles;
3. Thrombin augmentation:1000 units; and /or
4. N-butyl Cyanoacrylate (NBCA) embolotherapy (Glubran)

A total of 6 combinations of the above-mentioned embolic agents were utilized with the agents subsequently deployed via the microcatheter. These combinations included:

1. Coil embolization and thrombin injection: 4 mm coils were deployed in close proximity to the venous drainage site, followed by injection of thrombin via the microcatheter (**Figure 4**);
2. Embozene particles, coil embolization and thrombin augmentation: embozene particles (Range: 500 – 700 um) were deployed via the microcatheter in close proximity to the bleeding site. This was followed by coil embolization (4 to 5 mm coils). Subsequently, thrombin was injected via the catheter;
3. Embozene particles and thrombin augmentation: embozene particles (Range 500 – 700 um) were deployed via the microcatheter in close proximity to the bleeding site followed by injection of thrombin via the microcatheter;
4. Embozene particles and coil embolization: embozene particles (Range: 500 – 700 um) were deployed via the microcatheter in close proximity to the bleeding site. This was followed by coil embolization (4 to 5 mm coils);
5. NBCA embolotherapy alone: a plastic 3-way stopcock with two 5 cc non polycarbonate luer lock syringes were used to create a radiopaque mixture of NBCA with lipiodol (low dilution 1 NBCA :3 lipiodol mixture). The microcatheter was flushed with dextrose solution and subquently the NBCA/lipiodol mixture was administered via the microcatheter, with the catheter positioned close to the lesion

in a continuous column injection manner. Post embolization, the microcatheter was again flushed with dextrose and then removed. Post embolization angiogram was performed via the C2 glidecatheter **(Figure 3);** or

6. NBCA embolotherapy and Coil embolization: Two 4 mm coils were deployed upstream to the site of venous drainage responsible for the bleed. Then, the microcatheter was negotiated distal to the deployed coils and a NBCA/lipiodol combination was used to embolize the variceal bleeding site with the technique mentioned above. The microcatheter was subsequently removed and venogram performed via the C2 catheter demonstrated interval resolution of variceal bleed.

A post embolization angiogram was performed after each embolization, confirming resolution of the actively bleeding varix. Lastly, contrast injection via the originally placed sheath was performed to demonstrate a patent SMV, main, left and right portal venous branches. The determining factor of technical success was non-opacification/resolution of blood flow in the varices (**Figure 5, Figure 6**).

Supplementary Appendix 2

A total of twelve patients who underwent seventeen PATVO interventions for parastomal or small bowel EVs between September 2016 to September 2021 were identified **(Table S1)**. Five patients had repeat PATVO procedures within the review timeline. The mean age at intervention was 60.1 years (SD, 17.5), and 75% of patients were male (n=9). The clinical indication for the procedure in all patients was active or recurrent bleeding from parastomal or small bowel EVs.

Eleven patients had portal hypertension visualized on contrast enhanced CT imaging. Underlying hepatic etiology was present in eleven patients (91.7%). Of those 11 patients, four presented with non-alcoholic steatohepatitis associated cirrhosis, one patient presented with cirrhosis secondary to alcohol use, one patient presented with liver cirrhosis secondary to hepatitis C, one patient had prior hepatectomy with colon cancer metastases and one patient had a remote liver transplant with no evidence of acute or chronic rejection, and three patients presented with unspecified liver cirrhosis. The mean MELD score prior to intervention was 12.3 (SD, 4.2), and the mode Child-Pugh score was 7).

Eleven patients underwent a stoma formation at a mean age of 57.1 years (SD, 17.6) (n=10; age of one patient at stomal formation was not available). Among the patients with a stoma, two patients underwent prior surgical interventions for EV bleeding (25%). Among these surgical interventions, one patient underwent skin level surgical revision of ileostomy, and the second patient underwent skin level and full thickness surgical revisions of ileostomy. Both cases resulted in clinical failure of surgical intervention with subsequent re-bleeding from EVs prior to an interventional radiology referral. The above-mentioned surgical cases were subsequently managed with PATVO. The mean time interval between stoma formation and PATVO for those that underwent a stoma formation was 8.6 years (SD, 9.9) (n=10).

Bleeding secondary to parastomal EVs was identified in 10 patients (83%), whereas bleeding secondary to small bowel EVs was identified in two patients (17%) **(Table S2).** Among the parastomal EVs (n=10), four patients (40%) presented with bleeding from the right lower quadrant ileostomy site and six patients (60%) presented with bleeding from the left lower quadrant ileostomy site. Concomitant gastroesophageal varices were identified in five patients (41.7%) and concomitant portal vein thrombosis in one patient (8%). Nine EVs (75%) were supplied by branches of the SMV with portosystemic draining channels arising from either a separate branch of the SMV (44.4%; n=4), bilateral common iliac veins (11.1%; n=1), right common iliac vein

(11.1%; n=1), gonadal veins (11.1%; n=1), left portal vein (11.1%; n=1), or right common femoral vein (11.1%; n=1). The remaining three EVs (25%) were supplied by branches of the inferior mesenteric vein (IMV).

Table S1. Patient demographics and clinical data.

Case	Age at PATVO Intervention (SD)	Sex (%)	Clinical Indication (%)	Under-lying Liver Disease (%)	MELD Score (SD)	Child-Pugh Score	Age at Stoma Formation (SD)	Reason for Stoma Formation	Prior Surgical Reinterventions for Variceal Bleed	Time Interval from Stoma Formation to PATVO, in years (SD)
**Mean / Mode**	60.1 (17.5)[1]	Male (75)[1]	Stomal bleeding (65)[2]	Non-alcoholic steatohepatitis associated cirrhosis (33.3)[1]	12.3 (4.2)[1]	7[1]	57.1 (17.6)[1]	-	None	8.6 (9.9)[2]
**1**	47	Female	Stomal bleeding	Liver cirrhosis secondary to hepatitis C	11	7 (Class B)	39	Crohn Disease	(1) Skin level revision of ileostomy	8
**2**	61	Male	Stomal bleeding	Liver cirrhosis secondary to alcohol use	9	8 (Class B)	57	History of perforated appendix complication by abscess and anastomic leak requiring laparotomy, drainage of abscess, and ileocolic resection with ileostomy	NR	3
**3**	59	Male	Stomal bleeding	Prior hepatectomy with colon cancer metastases	14	9 (Class B)	57	Hartmann procedure due to locally advanced metastatic sigmoid carcinoma	None	1
**4**	53	Female	Stomal bleeding	Non-alcoholic steatohepatitis associated cirrhosis	8	5 (Class A)	52	Small intestinal angiodysplasia with recurrent GI hemorrhage, treated with small intestinal resection	None	1
**5**	23	Male	Stomal bleeding	Remote liver trans-	11	7 (Cla	Did not have an	N/A	None	N/A

Case	Age at PATVO Intervention (SD)	Sex (%)	Clinical Indication (%)	Under-lying Liver Disease (%)	MELD Score (SD)	Child-Pugh Score	Age at Stoma Formation (SD)	Reason for Stoma Formation	Prior Surgical Reinterventions for Variceal Bleed	Time Interval from Stoma Formation to PATVO, in years (SD)
				plantation and portal hypertension		ss B)	ostomy.			
**5***	23	Male	Stomal bleeding	Remote liver trans-plantation and portal hypertension	11	7 (Class B)	Did not have an ostomy.	N/A	None	N/A
**6**	93	Male	Stomal bleeding	Liver cirrhosis, specifics not available	7	7 (Class B)	NR	Total proctolectomy and end ileostomy for familial polyposis	NR	NR
**6***	93	Male	Recurrent stomal bleeding due to feeder vessel being missed at initial embolization	Liver cirrhosis, specifics not available	7	7 (Class B)	NR	Total proctolectomy and end ileostomy for familial polyposis	NR	NR
**7**	65	Female	Stomal bleeding	Non-alcoholic steatohepatitis associated cirrhosis	8	5 (Class A)	59	Recurrent colovesical fistula from diverticular disease	None	6
**7***	65	Female	Stomal bleeding	Non-alcoholic steatohepatitis associated cirrhosis	8	5 (Class A)	59	Recurrent colovesical fistula from diverticular disease	None	6
**8**	60	Male	Stomal bleeding	None	17	6 (Class A)	36	Ulcerative Colitis and later diagnosed with small bowel Crohn's disease	(1) skin level and (2) full thickness revision of ileostomy	24
**8***	61	Male	Recurrent chronic bleeding	None	17	6 (Class A)	36	Ulcerative Colitis and later diagnosed with small bowel	(1) skin level and (2) full thickness revision of ileostomy	24

Case	Age at PATVO Intervention (SD)	Sex (%)	Clinical Indication (%)	Under-lying Liver Disease (%)	MELD Score (SD)	Child-Pugh Score	Age at Stoma Formation (SD)	Reason for Stoma Formation	Prior Surgical Reinterventions for Variceal Bleed	Time Interval from Stoma Formation to PATVO, in years (SD)
**9**	59	Male	Stomal bleeding	Non-alcoholic steatohepatitis associated cirrhosis	17	7 (Class B)	29	Crohn's disease Crohn Disease	None	30
**10**	66	Male	Recurrent chronic bleeding	Non-alcoholic steatohepatitis associated cirrhosis	18	5 (Class A)	64	Complicated sigmoid diverticulitis	None	1
**10***	65	Male	Recurrent chronic bleeding	Non-alcoholic steatohepatitis associated cirrhosis	10	5 (Class A)	64	Complicated sigmoid diverticulitis	None	1
**11**	72	Male	Recurrent chronic bleeding	Liver Cirrhosis, specifics not available	9	5 (Class A)	71	Complicated sigmoid diverticulitis	None	1
**12**	83	Male	Recurrent chronic bleeding	Liver Cirrhosis, specifics not available	19	11 (Class C)	75	Metastatic colon cancer	None	7

Abbreviations: N/A, not applicable; NR, not reported; PATVO, percutaneous antegrade transhepatic venous obliteration; SMV, superior mesenteric vein.

*Patients 5, 6, 7, 8 and 10 underwent repeat PATVO interventions within the review time frame.

[1]Average is calculated per patient.

[2]Average is calculated per procedure.

Table S2. Anatomic considerations.

Case	Location of varices	Presence of gastroesophageal varices	Portal vein thrombosis	Supply of the varices
**Mode**	Parastomal: LLQ Ileostomy site	No	No	Supplied by two separate branches of SMV
**1**	Parastomal: LLQ Ileostomy site	No	No	Supplied by SMV with collateral supply with bilateral common iliac veins
**2**	Parastomal: RLQ Ileostomy site	No	No	Supplied by SMV with collateral supply with right common iliac vein
**3**	Parastomal: RLQ colostomy site	No	Yes	Supplied by two separate branches of SMV
**4**	Small bowel at the region of ileum	No	No	Supplied SMV with collateral supply with gonadal veins
**5**	Small bowel in the proximal jejunum	Yes	No	Supplied by SMV draining with collateral supply with left portal vein
**5***	Small bowel in the proximal jejunum	No	No	Supplied by SMV draining with collateral supply with left portal vein
**6**	Parastomal: LLQ Ileostomy site	No	No	Supplied by two separate branches of SMV
**6***	Parastomal: LLQ Ileostomy site	No	No	CT demonstrates an additional branch of the SMV supplying the varix
**7**	Parastomal: LLQ Ileostomy site	Yes	No	Supplied by two separate branches of SMV
**7***	Parastomal: LLQ Ileostomy site	No	No	Supplied by branches of the IMV
**8**	Parastomal: RLQ Ileostomy site	Yes	No	Supplied by two separate branches of SMV
**8***	Parastomal: RLQ Ileostomy site	No	No	Supplied by two separate branches of SMV
**9**	Parastomal: RLQ Ileostomy site	Yes	No	Supplied by branches of SMV with collateral supply with right common femoral vein
**10**	Parastomal: LLQ Ileostomy site	No	No	Supplied by branches of the IMV
**10***	Parastomal: LLQ Ileostomy site	No	No	Supplied by branches of the IMV
**11**	Parastomal: LLQ Ileostomy site	No	Yes	Supplied by branches of the IMV and portal vein
**12**	Parastomal: LLQ Ileostomy site	Yes	No	Supplied by branches of the IMV

Abbreviations: CT, Computed Tomography; LLQ, left lower quadrant; RLQ, right lower quadrant; SMV, superior mesenteric vein; IMV, inferior mesenteric vein.

*Patients 5, 6, 7, 8 and 10 underwent repeat PATVO interventions within the review time frame.
